# Transcriptome profiling of transcription factors in *Ganoderma lucidum* in response to methyl jasmonate

**DOI:** 10.3389/fmicb.2022.1052377

**Published:** 2022-11-24

**Authors:** Xiaolan Xu, Fengli Zhu, Yuxuan Zhu, Yujie Li, Hao Zhou, Shilin Chen, Junshan Ruan

**Affiliations:** ^1^College of Animal Sciences (College of Bee Science), Fujian Agriculture and Forestry University, Fuzhou, China; ^2^College of Food Science, Fujian Agriculture and Forestry University, Fuzhou, China; ^3^Institute of Chinese Materia Medica, China Academy of Chinese Medical Sciences, Beijing, China; ^4^Institute of Herbgenomics, Chengdu University of Traditional Chinese Medicine, Chengdu, China; ^5^Fujian Provincial Hospital, Fuzhou, China

**Keywords:** *Ganoderma lucidum*, ganoderma triterpenoid, UPLC/Q-TOF-MS/MS, methyl jasmonate, *Glmhr*, transcription factors

## Abstract

*Ganoderma lucidum* is a traditional Chinese medicine and its major active ingredients are ganoderma triterpenoids (GTs). To screen for transcription factors (TFs) that involved in the biosynthetic pathway of GTs in *G. lucidum*, the chemical composition in mycelia, primordium and fruiting body were analyzed, and the transcriptomes of mycelia induced by methyl jasmonate (MeJA) were analyzed. In addition, the expression level data of MeJA-responsive TFs in mycelia, primordia and fruiting body were downloaded from the database, and the correlation analysis was carried out between their expression profiles and the content of total triterpenoids. The results showed that a total of 89 components were identified, and the content of total triterpenoids was the highest in primordium, followed by fruiting body and mycelia. There were 103 differentially expressed TFs that response to MeJA-induction including 95 upregulated and 8 downregulated genes. These TFs were classified into 22 families including C2H2 (15), TFII-related (12), HTH (9), fungal (8), bZIP (6), HMG (5), DADS (2), etc. Correlation analysis showed that the expression level of GL23559 (MADS), GL26472 (HTH), and GL31187 (HMG) showed a positive correlation with the GTs content, respectively. While the expression level of GL25628 (fungal) and GL26980 (PHD) showed a negative correlation with the GTs content, respectively. Furthermore, the over expression of the *Glmhr1* gene (GL25628) in *Pichia pastoris* GS115 indicated that it might be a negative regulator of GT biosynthesis through decreasing the production of lanosterol. This study provided useful information for a better understanding of the regulation of TFs involved in GT biosynthesis and fungal growth in *G. lucidum*.

## Introduction

*Ganoderma lucidum*, also named “Lingzhi,” has been used as a traditional Chinese medicine with the effects of improving health and promoting longevity for thousands of years in China. The chemical constituents of *G. lucidum* are very complex, including triterpenoids, polysaccharides, nucleosides, furan derivatives, sterols, alkaloids and polypeptides (Ren et al., 2010; Li et al., 2013; Xie et al., 2020; Dong et al., 2021). Among them, triterpenoids are one of the most important bioactive components, exhibiting antitumor, apoptotic, antiviral and immune-modulatory pharmaceutical activities (Chi et al., 2018; Liang et al., 2019; Shao et al., 2021; Chen et al., 2022a). Ganoderma triterpenoids (GTs) are derived from lanosterol, which is the intermediate of triterpenoids and synthesized by lanosterol synthase (LSS) through the mevalonate (MVA) pathway. Currently, approximately 150 GTs have been identified in *G. lucidum*, such as ganoderic acids, methyl ganoderate, lucidenic acid, and ganolucidic acid (Chen et al., 2012; Xie et al., 2020).

Triterpenoids concentration in aerial mycelia is minimal, but it may be raised by liquid fermentation, which is influenced by the growth conditions and medium used. To improve the production of GT, a few biotic and abiotic elicitors had been used to enhance the GT production, such as ozone gas, nitric oxide, ozone gas, methyl jasmonate (MeJA), and salicylic acid (SA) (Ren et al., 2010; Shi et al., 2015; Sudheer et al., 2016; Gu et al., 2017; Liu et al., 2018). JA and its derivatives MeJA, which have been used widely as important elicitors, are effective in inducing the production of secondary metabolites in many plant and fungal species (Wang et al., 2014; Ali et al., 2015; Kim et al., 2017; Zhou et al., 2019; Lin et al., 2020; Chen et al., 2022b). In *G. lucidum*, MeJA also has been confirmed as an effective elicitor for the induction of GT via an ROS signaling pathway (Shi et al., 2015). Except for the induction of secondary metabolites, the MeJA induction also affects plant and fungal stress resistance, growth development, external morphology and other physiological processes (Aranega-Bou et al., 2014).

Secondary metabolites are all biosynthesized through metabolic pathways, which are controlled by a complex regulatory network that regulated by transcription regulators. Many transcription factors (TFs), which participate in JA- and MeJA-induced secondary metabolite biosynthesis, could upregulate or downregulate the expression of genes involved in metabolism biosynthesis (Wang et al., 2014; Ali et al., 2015; Kim et al., 2017; Zhou et al., 2019; Lin et al., 2020; Chen et al., 2022b). A few genome-wide transcriptome analyses have revealed that JA and MeJA could extensively regulate TFs to enhance the production of secondary metabolites including terpene, taxol, nicotine, and flavonoid. These MeJA- responsive TFs include the ORCA, ERF, MYC, WRKY, bHLH, MYB, and bZIP families (Tsubasa and Takashi, 2011; Yu et al., 2012; Afrin et al., 2015; Lin et al., 2020; Chen et al., 2022b). For example, the bHLH TFs positively regulated the terpenoid indole alkaloid biosynthesis in *Catharanthus roseus* and the nicotine biosynthesis in *Nicotiana benthamiana* (Suttipanta et al., 2011; Zhang et al., 2011). The identification of MeJA-responsive TFs by transcriptome analysis is an effective method to screen for candidate genes involved in metabolism biosynthesis pathway and provide useful information to elucidate the transcriptional regulation of a particular biosynthetic pathway.

The functions of many TFs in plants and fungi have been verified. *G. lucidum* was found to have approximately 600 TFs in the whole genome (Chen et al., 2012), but until now, a few TFs, such as *GlPacC*, *GlSwi6*, *GlSkn7*, and *CRZ1*, have been reported (Liu et al., 2013; Wu et al., 2016; Wang et al., 2018; Zhang et al., 2018; Hu et al., 2019; Li and Zhong, 2020). These TFs were involved in the growth process of *G. lucidum*, and their silencing resulted in the changes of the content of ganoderma acids (Liu et al., 2013; Wang et al., 2018; Zhang et al., 2018). In *G. lucidum*, the function of a large number of TFs is still unclear. Consequently, the understanding of the transcriptional regulation in this fungus is limited. In this study, the chemical components in different growth phases and the transcriptome profile *G. lucidum* were analyzed to screen TFs that possibly be involved in the synthesis of triterpenoids. Furthermore, the over expression of *Glmhr1* (GL25628), a GAL4-like TF, in *Pichia pastoris* GS115 revealed that it was negative correlation with GTs yield in *G. lucidum.* This study provided information about the roles of TFs in the GTs biosynthesis and fungal growth.

## Materials and methods

### Chemical analysis

The primordia and fruiting body (fruiting body formation stage) of *G. lucidum* G203 were collected from Jianning, Fujian province in China. Aerial mycelia were collected by culturing on potato dextrose agar (PDA) plates with glass paper at 28°C for 10 days. All the samples were ultrasonic extracted using the method as described in “Chinese pharmacopoeia 2020.” Samples (2 g) were extracted with ethanol (50 ml) by ultrasonic method with the parameters of 140 W and 42 kHz for 45 min. The extracts were filtered and adjusted to 100 ml using ethanol. The ethanol extracts of samples were used for the detection of total triterpenoids and chemical composition. To create a standard curve for determining the content of total triterpenoids, 0.0, 0.2, 0.3, 0.4, 0.5, and 0.6 ml of oleanolic acid (0.2 mg/ml) were added 5% vanillin-glacial acetic acid solution (0.2 ml) and perchloric acid (0.8 ml), respectively. The mixtures were heated in the 70^°^C water bath for 15 min, and then reacted with ethyl acetate solution (4 ml). The absorbance of the samples were measured at 560 nm and the standard curve obtained is y = 8.6735 x − 0.0073 (*R*^2^ = 0.9993).

The extracts of samples were filtered through 0.22 μm filter membrane for constituent analysis. Analyses were carried out on a Waters UPLC system using an ACQUITY UPLC HSS T3 column (2.1 × 100 mm, 1.7 μm) at a flow rate of 0.3 mL/min. The mobile phases A and B consisted of 0.1% formic acid in water and acetonitrile, respectively. The mobile phase gradient program was as follows: 0–1 min, 100% A; 1–35 min, 100% A–50% A; 35–50 min, 50% A–20% A; 50–55 min, 20% A–0% A; 55–57 min, 0% A, 57.1–60 min, 100% A. The on-line UV spectra were scanned from 200 to 400 nm.

The mass spectra were acquired in both positive and negative ion modes by using a Waters definition accurate mass quadrupole time-of-flight (Q-TOF) Xevo G2-XS mass spectrometer (Waters, Milford, MA, USA) equipped with an ESI source. The optimized operating parameters were as follows: mass range, m/z 50–1,500; the flow rate of drying gas (N_2_), 800 L/h; drying gas temperature, 400^°^C; cone gas flow, 100 L/h; source temperature, 120^°^C; capillary voltage, 2.5 kV; cone voltage, 40 V; In MSe mode, the energies for collision induced dissociation (CID) were 6 V for the precursor ion at low energy mode and 20–60 V for fragmentation information at high energy mode. An external reference (Lock-Spray TM) consisting of a 0.2 ng/mL solution of Leucine enkephalin was used in both positive (m/z 556.2771 [M + H]^+^) and negative mode (m/z 554.2615 [M–H]^–^), infused at a flow of 5 μL/min. All the data was acquired using Mass Lynx TM 4.1 software (Waters, Milford, MA, USA).

### Strains culture and methyl jasmonate treatment

The monokaryotic *G. lucidum* strain G.260125-1, whose whole-genome had been sequenced, was used for transcriptome analysis. The mycelia of *G. lucidum* strain G.260125-1 were treated with MeJA according to the previously reported method (Shi et al., 2015). After the strain was cultured on PDA plates at 28°C for 7 days, the mycelia were cultured in potato-dextrose broth with MeJA at final concentrations of 0, 50, 100, 150, 200, and 300 μM at 28°C and 150 rpm for 7 days in a shock incubator. The mycelia treated with equal volume of solvent without MeJA were used as control. Thereafter, the biomass and the contents of total triterpenoids were determined. Each treatment and all determinations were carried out in triplicate.

### Transcriptome analysis

Total RNA was extracted from mycelia that treated with MeJA at final concentrations of 0, 200, and 300 μM using Trizol reagent (Invitrogen, Carlsbad, CA, USA) according to the manufacturer’s protocol. RNA quantity and quality were evaluated with a NanoDrop 2000 spectrophotometer (Thermo Fisher Scientific, MA, USA) and an Agilent 2100 Bioanalyzer (Agilent Technologies, Santa Clara, CA). The samples whose RNA Integrity Number (RIN) values were greater than 8.5 were used for further experiment. And mRNA was isolated from total RNA using poly- (T) oligo- linked magnetic beads. All isolated mRNA was sheared into short fragments (150–200 nt) by adding fragmentation buffer and used for first strand cDNA synthesis with random primers. The second strand cDNA were synthesized and purified with a QIAquick PCR Purification Kit (Qiagen, Dusseldorf, Germany). After the purified cDNAs were end repaired and acetylated at their 3′ ends, the pair-end adapters were connected. At last, the cDNA libraries were verified using an Agilent 2100 Bioanalyzer and ABI StepOnePlus Real-time PCR system and sequenced using an Illumina HiSeqTM 4000 (Illumina Inc., Delaware, USA). All the raw data have been submitted to the NCBI database (Accession No: PRJNA865720).

Raw data in fasta format were processed using Perl scripts, and clean data were obtained by removing adapter sequences, reads containing more than 10% “N” rate and low-quality reads. The clean reads were mapped to the genome sequence of *G. lucidum* G.260125-1 (Accession No: AGAX00000000.1) using Bowtie2 software. The genes that were quantitatively analyzed using RSEM and Noiseq software were used to screen the differentially expressed genes (DEGs) between different treatment groups according to the fragments per kilobase of transcript sequence per millions base pairs sequenced (FPKM) method. The genes with |log2FC| ≥ 1 and probability value >0.8 were considered as DEGs.

### Verification of quantitative real-time PCR

Total RNA was extracted using Magnetic bead Method Universal RNA Extraction Kit (Thermo Fisher Scientific, MA, USA) according the manufacturer’ procedure. Residual genomic DNA was removed, and 500 ng of RNA without genomic DNA was reverse-transcribed for quantitative real-time PCR (qRT-pCR) according the manufacturer’s procedure (Takara Bio, Kyoto, Japan). The gene transcription levels of different treatment groups were quantified by qRT-PCR. The primer sequences are shown in Table 1. The reaction procedure was performed on CFX 384 Real-Time System C1000 Touch Thermal Cycler (BioRad, California, USA) using a two-step method as follows: 95°C for 30 s, 40 cycles of 95°C for 5 s, and 60°C for 30 s. Transcript level of genes was calculated according to the 2^–△△*CT*^ method and *G. lucidum* glyceraldehyde-3-phosphate dehydrogenase (*GL-GPD*) gene transcripts were used as an internal control.

**TABLE 1:** Primer sequences used for qRT-PCR analysis.

Primers	Sequences (F)	Sequences (R)
GPD	GATGAAGGACTGGCGTGGT	CCGTTGAGGCTGGGAATGAC
GL22438	GGAAAGGGGAAAGGTGTC	CGTTGGCGAGGGAGAAG
GL29539	CCCTTACGAGGTGTCCG	GCGACTGGTCCATCTGC
GL22204	TACAAGACCATCCTCAAC	GCTCTCCTTCTTCTGATAT
GL25456	TATCTGCTATCGTCCATT	CCCTTGACTTGATCTATG
GL25655	GTCTTCGCCTCTATATCC	AGATACTTCAGGACTCAAG
GL31187	CTCCACCCAGAACCCAAG	GGATTGAAGACATAGAACTGTAGT
GL20710	AATCTTCAGCAGCAGGTC	TTGGTGTTGACTTGTCTGT
GL19576	AATCGGCTCTATCCTTGG	GTAGGCATAACAGTCACC
GL24137	TCCAAGTGGCGTCAAGGT	TCATCGTCTTCTTCATCGTCTTCA

### Over expression of *Glmhr1* gene

The over expression of *Glmhr1* gene (GL25628) in *P. pastoris* GS115 were performed according to the method that described in previous study (Ren et al., 2012). RNA was isolated and reverse transcription was carried out using the PrimeScript RT Master Mix kit (Takara Bio, Kyoto, Japan). *Glmhr* cDNA was amplified from *G. lucidum* using the forward primer 5′-GAATTCATGGCCGAGGAGCGGAAACCCT-3′ and the reverse primer 5′-GCGGCCGCCTAGCCCAACA AGACTTGAAA-3′. Purified PCR product was connected with T vector (Takara Bio, Kyoto, Japan) and the plasmid was transformed into the competent *Escherichia coli* DH 5α cells. The positive colony was verified by PCR and sequencing.

The verified *Glmhr* cDNA fragment was digested with *Xho*I and *Not*I enzymes, and the digested fragment was ligated into the *Xho*I/*Not*I-digested pPIC9k to construct the recombinant plasmid pPIC/Glmhr1, which was then transformed into the competent *E. coli DH* 5α cells. The positive colony was verified by PCR and sequencing. The plasmid pPIC/Glmhr1 was digested by *Sal*I, and then purified by agarose gel electrophoresis. The linearized plasmids were transformed into the competent *P. pastoris* GS115 cells by electroporation under 1,500 V. The positive colony were selected according to the method that described in previous study (Buensanteai et al., 2010), and then verified by PCR using the primers 5′-GCAAATGGCATTCTGACATCC-3′ and 5′-GACTGGTTCCAATTGACAAGC-3′. And the positive strain was named as GS115/Glmhr1.

The strain GS115/Glmhr1 was grown in 50 ml of BMGY medium for about 20 h at 30^°^C and 200 rpm. When the OD600 nm reached at about 4.0–8.0, cells were centrifuged and then resuspended and inoculated in 100 ml of BMMY medium. The yeasts were cultured for 5 days and 100% methanol was added every 24 h to the final concentration of 0.5%. After 5 days culture, the cells were collected and the expression of recombinant proteins was analyzed by western blot, and the contents of lanosterol and ergosterol were determined.

### *In vitro* lanosterol and ergosterol content determination assay

Yeast strains GS115/Glmhr1 and GS115 cultures were centrifuged at 7,000 g for 10 min and resuspended twice in sterile distilled water to remove culture media. The yeasts were freeze-dried and treated with 10% KOH-methanol solution in 90^°^C water bath for 2 h, then equal volume of n-hexane was added. The n-hexane extracts were used to determine the contents of lanosterol and ergosterol. Analyses were carried out on an Agilent HPLC system using an Eclipse Pluse C18 (2.1 × 50 mm, 1.8 μm). The mobile phases A (95%, v/v) was 0.1% acetic acid in water and B (5%, v/v) was methanol. The flow rate was 0.2 mL/min, and the detection wavelength was 210 nm.

### Statistics analysis

The correlation analysis between the contents of total triterpenoids and the expression profiles of TFs were carried out using SPSS version 20.0 (SPSS Inc., Chicago, IL, USA). The data mean ± standard error is calculated based on the results of three technical replicates using SPSS version 20.0 (SPSS Inc., Chicago, IL, USA). Experimental data was analyzed by One-way ANOVA followed by a *post-hoc* Tukey test, and P < 0.05 was considered statistically significant.

## Results

### Chemical analysis of *Ganoderma lucidum*

**FIGURE 1:**
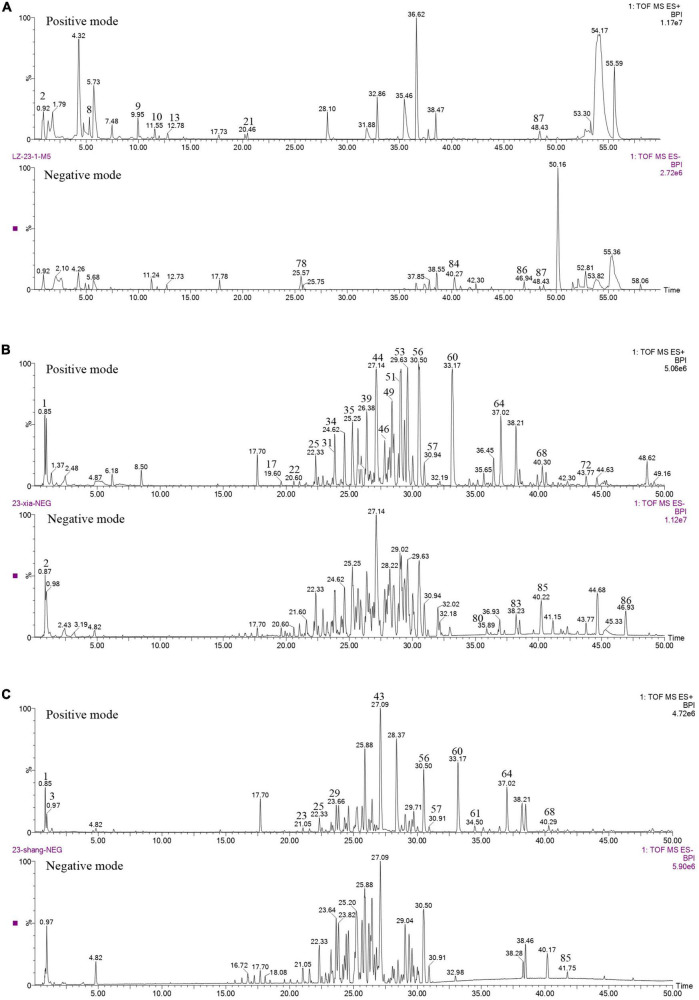
Chemical analysis of *G. lucidum* in different growth periods. **(A)** Aerial mycelia, **(B)** primordium, **(C)** fruiting body.

The content of total triterpenoids was the highest in primordia (15.77 mg/g) followed by fruiting body (13.52 mg/g) and aerial mycelia (6.44 mg/g), which is consistent with our previous study (Chen et al., 2012). The chemical composition in different periods were further analyzed by UPLC/Q-TOF-MS/MS. As shown in Figure 1, a total of 89 components were detected in mycelia, primordium and fruiting body, including triterpenoids, organic acids, alkaloids, fatty acids and flavonoids, among which triterpenoids were the most important components (Table 2). Most of the components were found both at the positive and negative poles, and some were detected only at the positive pole or at the negative pole (Supplementary Table 1). Only Ganoderic acid T was detected out in the mycelia, while there were 45 and 48 triterpenoids in the primordium and fruiting body, respectively. Of these triterpenoids, 37 were identified in both primordium and fruiting body, and contents of many triterpenoids in the primordia were higher than that in fruiting body formation.

**TABLE 2:** Chemical analysis of *G. lucidum* in different growth periods.

NO.	tR (min)	Identification	Source	Selected ion	Elemental composition	NO.	tR (min)	Identification	Source	Selected ion	Elemental composition
1	0.85	5-Methylcytidine	M, P, F	[M + H]^+^	C10H15N3O5	46	27.76	Resinacein G^#^	P, F	[M + H]^+^	C30H40O8
2	0.87	Gluconic acid	M, P, F	[M-H]^–^	C6H12O7	47	28.05	Lucideric acid A^#^	P, F	[M + H]^+^	C27H38O6
3	0.97	Sucrose	P, F	[M + HCOO]^–^	C12H22O11	48	28.19	Ganoleucoin E^#^	P, F	[M + H]^+^	C27H38O6
4	1.22	Malic acid	P, F	[M-H]^–^	C4H6O5	49	28.49	Ganoderenic acid D^#^	P, F	[M + H]^+^	C30H40O7
5	2.20	Citric acid	P	[M-H]^–^	C6H8O7	50	28.85	Lucidenic acid F^#^	P, F	[M + H]^+^	C27H36O6
6	3.83	Uridine	M, P, F	[M-H]^–^	C9H12N2O6	51	29.04	Gibbosic acid L^#^	P, F	[M + H]^+^	C30H40O6
7	5.07	Histidylprolinamide	P	[M + H]^+^	C11H17N5O2	52	29.35	Lucidenic acid D^#^	P, F	[M + H]^+^	C29H38O8
8	5.37	Phenylalanine	M, P	[M + H]^+^	C9H11NO2	53	29.63	Ganoderic acid E^#^	P, F	[M + H]^+^	C30H40O7
9	9.95	hexahydro-7-hydroxy-3-[(1R) –1–methylpropyl]-Pyrrolo[1,2-a]pyrazine-1,4-dione	M	[M + H]^+^	C11H18N2O3	54	29.99	Ganolucidic acid D^#^	P, F	[M + H]^+^	C30H44O6
10	11.55	L-Leu-L-Phe	M	[M + H]^+^	C14H16N2O3	55	30.13	Ganodernoid D^#^	F	[M + H]^+^	C32H40O9
11	11.67	Riboflavin	P, F	[M + H]^+^	C17H20N4O6	56	30.50	Ganoderic acid F^#^	P, F	[M + H]^+^	C32H42O9
12	11.99	Cyclo(pro-leu)	M	[M + H]^+^	C11H18N2O2	57	30.94	Ganoderic acid LM2^#^	P, F	[M + H]^+^	C30H42O7
13	12.78	Cyclo(L-IIe-L-Pro)	M	[M + H]^+^	C11H18N2O2	58	31.27	12β-Acetoxy-3,7,11,15,23-pentaoxolanosta-8,20-dien-26- oic acid^#^	P, F	[M + H]^+^	C32H40O9
14	14.33	Cyclo(L-Phe-L-Pro)	M	[M + H]^+^	C14H16N2O2	59	32.27	Ganoderiol D^#^	P, F	[M + H]^+^	C30H48O5
15	15.87	Oregonensin A	F	[M-H]^–^	C16H16O6	60	33.17	Phytosphingosine	P, F	[M + H]^+^	C18H39NO3
16	16.99	Hesperidin	P	[M + H]^+^	C28H34O15	61	34.51	Ganoderitriol M^#^	P, F	[M + H]^+^	C30H50O4
17	19.0	12-Hydroxyganoderic acid C2^#^	P, F	[M + H]^+^	C30H46O8	62	35.29	*C*19-Phytosphingosine	M, P, F	[M + H]^+^	C19H41NO3
18	19.91	Applanatumol S	F	[M-H]^–^	C16H20O6	63	36.04	Ganolucidic acid E^#^	P	[M + H]^+^	C30H44O5
19	20.06	Sebacic acid^#^	P, F	[M-H]^–^	C10H18O4	64	37.02	C20-Phytosphingosine	P, F	[M + H]^+^	C20H43NO3
20	20.20	α-Solanine	M	[M + H]^+^	C45H73NO15	65	37.17	C20-Phytosphingosine	P, F	[M + H]^+^	C20H43NO3
21	20.46	Chaconine	M	[M + H]^+^	C45H73NO14	66	39.72	24,25-epoxy-26,27-dihydryoxy-Lanosta-7,9(11)-dien-3-one^#^	F	[M + H]^+^	C30H46O4
22	20.60	Ganoderic acid L^#^	P, F	[M + Na]^+^	C30H46O8	67	40.17	Linolenic acid	M	[M + H]^+^	C18H30O2
23	21.05	Ganoderic acid AP^#^	P	[M + H]^+^	C30H42O9	68	40.30	2-Amino-1,3-docosanediol	P, F	[M + H]^+^	C22H47NO2
24	21.60	(3β,7β,25R)-3,7,20,24-tetrahydroxy-11,15,23-trioxo-Lanost-8-en-26-oic acid^#^	P	[M + H]^+^	C30H44O9	69	41.13	Ganodermaside D	F	[M + H]^+^	C28H40O2
25	22.33	Ganoderic acid G^#^	P, F	[M + H]^+^	C30H44O8	70	41.78	9-Hydroxy-(10E,12Z,15Z)-octadecatrienoic acid^#^	M, P, F	[M + H]^+^	C18H30O3
26	22.53	Ganoderic acid C6^#^	P, F	[M + H]^+^	C30H42O8	71	41.91	Ganoderiol E^#^	F	[M + Na]^+^	C30H48O4
27	22.89	3β,7β,15α,24-tetrahydroxy-11,23-dioxo-5α-lanosta-8,20E-dien-26-oic acid^#^	P, F	[M + H]^+^	C30H44O8	72	43.79	Ganoderic acid DM^#^	P, F	[M + H]^+^	C30H44O4
28	23.24	Ganoderenic acid C^#^	P, F	[M + H]^+^	C30H44O7	73	45.15	Ganodermanondiol^#^	P	[M + H]^+^	C30H48O3
29	23.66	Ganoderic acid H^#^	P, F	[M + H]^+^	C32H44O9	74	47.34	Demethylincisterol A3	M	[M + H]^+^	C21H32O3
30	23.80	Ganoderic acid C2^#^	P, F	[2M + H]^+^	C30H46O7	75	48.46	9(11)-Dehydroergosterol	M	[M + H]^+^	C28H42O
31	24.17	Ganoderic acid J^#^	P	[M + H]^+^	C30H42O7	76	23.06	Lucidenic acid M^#^	P	[M-H]^–^	C27H42O6
32	24.29	Lucidenic Acid R^#^	P, F	[M + H]^+^	C32H44O10	77	24.40	Lucidenic acid N^#^	P, F	[M-H]^–^	C27H40O6
33	24.47	Ganolucidate F^#^	P, F	[M + H]^+^	C30H46O6	78	25.57	Pinellic acid	M	[M-H]^–^	C18H34O5
34	24.62	Resinacein H^#^	P, F	[M + H]^+^	C30H42O8	79	29.77	12-Acetoxy-3-hydroxy-7,11,15-trioxolanost-8,16,24-trien-26-oic acid^#^	P	[M-H]^–^	C32H42O8
35	25.25	7β,15α,20-Trihydroxy-3,11,23-trioxo-5α-lanosta-8-en-26-oic Acid^#^	P, F	[M + H]^+^	C30H44O8	80	35.89	Elfvingic acid B^#^	F	[M-H]^–^	C30H40O8
36	25.65	Ganoderic acid B^#^	P, F	[M + H]^+^	C30H44O7	81	36.89	7-Oxoganoderic acid Z^#^	F	[M + HCOO]^–^	C30H46O4
37	25.94	Ganoderic acid D^#^	P, F	[M + H]^+^	C30H42O7	82	37.30	Ganoderic Acid ZXYL^#^	F	[M-H]^–^	C30H46O5
38	26.19	Ganoderenic acid K^#^	P, F	[M + H]^+^	C32H44O9	83	38.23	Ganomycin I	F	[M-H]^–^	C21H26O4
39	26.38	Ganoderic acid C^#^	P, F	[M + H]^+^	C32H42O7	84	40.27	13 hydroxyoctadecadieno?ic acid	M, P, F	[M-H]^–^	C18H32O3
40	26.46	Spiroganocalitone D^#^	P, F	[M + H]^+^	C32H44O8	85	41.75	(11α)-11,26,27-trihydroxy-lanosta-8,24-diene-3,7-dione^#^	P	[M-H]^–^	C30H46O5
41	26.63	Ganoderic acid V1^#^	P, F	[M + H]^+^	C30H42O7	86	46.93	2-Hydroxy-hexadecanoic acid	M, P, F	[M-H]^–^	C16H32O3
42	26.79	(7β,12β,20Z)-12-(Acetyloxy)-7-hydroxy-3,11,15,23-tetraoxolanosta-8,20(22)-dien-26-oic acid^#^	P	[M + H]^+^	C32H42O9	87	48.43	Ganoderic acid T^#^	M	[M-H]^–^	C36H52O8
43	27.11	12β-Acetoxyganoderic Acid θ^#^	P	[M + H]^+^	C32H44O9	88	48.79	Palmitoleic acid	M	[M-H]^–^	C16H30O2
44	27.14	Ganoderic acid A^#^	P, F	[M-H_2_O + H]^+^	C30H44O7	89	51.57	2-Hydroxy-octadecanoic acid	M, P, F	[M-H]^–^	C18H36O3
45	27.39	Ganolucidic acid A^#^	P, F	[M + H]^+^	C30H44O6						

M, mycelia; P, primordium; F, fruiting body. ^#^GT.

### Methyl jasmonate–induced biomass and ganoderma triterpenoids production of the mycelia

**FIGURE 2:**
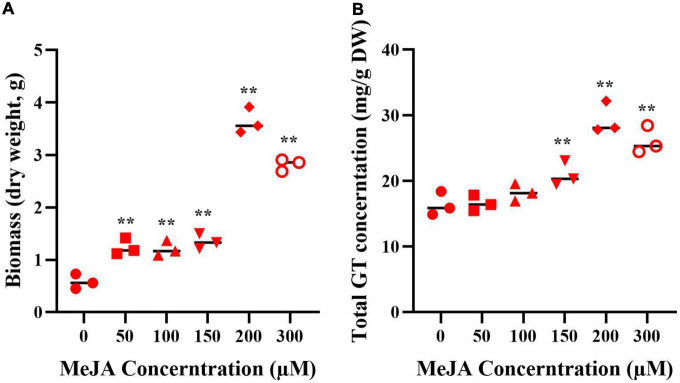
The biomass **(A)** and GT content **(B)** of *G. lucidum* in response to MeJA treatment. One-way analysis of variance (ANOVA) followed by a post-hoc Tukey test. ^**^Means extremely significant difference between treatment and control, *p*-value < 0.01.

The mycelia were treated with MeJA at final concentrations of 50, 100, 150, 200, and 300 μM, the fugal biomass (dry weight, DW) and the contents of total triterpenoids were shown in Figure 2. The biomass of mycelia with different concentration of MeJA treatment were 2.13, 2.09, 2.32, 6.26, and 4.87-fold greater than that of the control (Figure 2A), and total triterpenoids contents were 1.09, 1.23, 1.38, 2.17, and 1.93-fold higher (Figure 2B). The results showed that the biomass and the contents of total triterpenoids could be significantly increased through the induction of MeJA, and both peaked at 200 μM. Based on the change of biomass and the contents of total triterpenoids, the mycelia treated with MeJA at final concentrations of 0, 200, and 300 μM were used for transcriptome analysis.

### Identification of putative transcription factors involved in ganoderma triterpenoid synthesis

**FIGURE 3:**
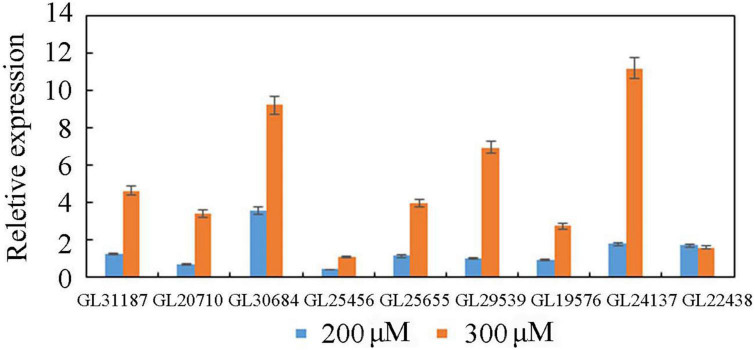
Quantitative real-time PCR analysis of TFs.

Three groups of RNA samples were used for the Illumina sequencing to obtain totals of 134577592, 133451088, and 132872034 clean reads. As a result, a total of 1,316 and 2,448 DEGs were identified in 200 μM treatment group and 300 μM treatment group, respectively (Supplementary Table 2). To validate the reliability of the RNA-Seq data, the expression level of 9 TFs was randomly selected for qRT-PCR assays (Figure 3). The results showed that the expression levels detected by qRT-PCR assay matched well with the RNA-Seq data, and the qRT-PCR data confirmed the reliability of the results in the RNA-Seq analysis.

**FIGURE 4:**
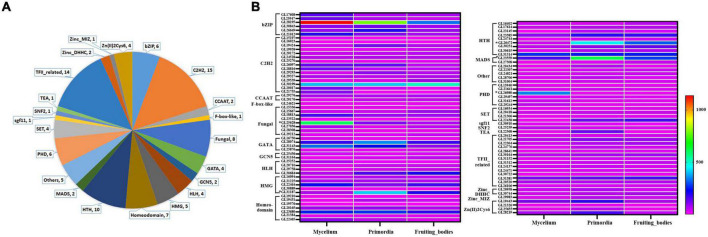
Classes analysis of differently expressed TFs and their expression level in mycelia, primordium and fruiting body. **(A)** Classes analysis; **(B)** expression level.

In the genome of *G. lucidum*, there are approximately 600 TFs, 103 of which were significantly differentially expressed in response to MeJA elicitation (Supplementary Table 3). These differently expressed TFs (DETs) are involved the fungal growth, secondary metabolism and stress responses, including C2H2 (18), TFII-related (15), HTH (12), fungal (9), bZIP (6), HMG (6), etc (Figure 4A). Among them, there were 39 DETs that detected in both 200 μM group and 300 μM treatment group. And the expression level of 25 DETs in 200 μM was higher than that in 300 μM, which was consistent with the change trend of the contents of triterpenoids and mycelia biomass after MeJA treatment. Besides, 8 TFs were down-regulated after MeJA induction.

Our group had reported the transcriptome of *G. lucidum* at different growth stages including mycelia, primordium and fruiting body (fruiting body formation stage) (Chen et al., 2012). The gene expression levels of 103 DETs in different periods were evaluated to more precisely screen the TFs that possibly be involved in the GT synthesis (Figure 4B and Supplementary Table 3). Overall, 19 TFs were highly expressed in mycelia (relative expression level > 100), 25 TFs in primordia and 15 TFs in fruiting body. Among them, 9 TFs (GL28195, GL30199, GL28074, GL22646, GL23680, GL23585, GL31314, GL23559, and GL31381) were highly expressed in mycelia, primordia and fruiting body periods, especially GL28195 (bZIP) and GL30199 (C2H2). Additionally, GL25628 (fungal) was especially highly expressed in mycelia, while GL26472 (HSF) was highly expressed in primordia and fruiting body.

Correlation analysis revealed that the expression profiles of 35 MeJA-responsive TFs were positively correlated with the contents of total triterpenoid during development [correlation coefficient (r) > 0.85] and 5 were negatively correlated with the contents of total triterpenoid (*r* < –0.85), suggesting they might be involved in triterpenoid biosynthesis (Supplementary Table 3). On the basis of the expression profiles of these 40 TFs, GL23559 (MADS), GL26472 (HTH), and GL31187 (HMG) were most likely to positively regulate GTs biosynthesis, while GL25628 (fungal) and GL26980 (PHD) were most likely to be negative regulators. Among them, *Glmhr1* (GL25628) was a candidate TF for regulating the expression of *FPS*, which is one of the important genes for lanosterol production through MVA pathway (Xu et al., 2020).

### The effect of *Glmhr1* on the lanosterol and ergosterol content

**FIGURE 5:**
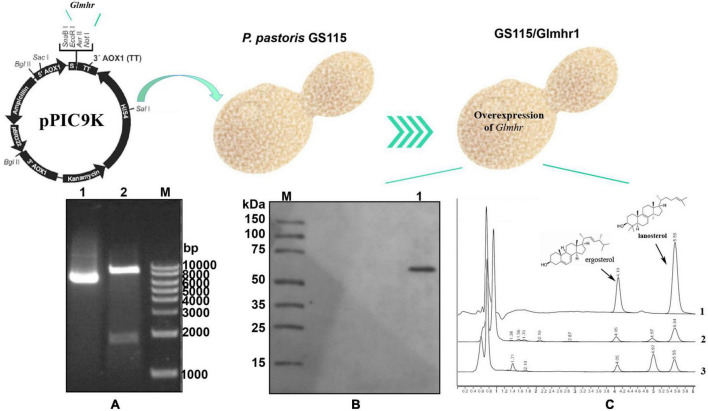
Over expression of Glmhr1 gene. **(A)** Verification of the recombinant plasmid pPIC9k/Glmhr1. M: Maker; 1: plasmid pPIC9k/Glmhr1; 2: *Xho*I/*Not*I- digested plasmid pPIC9k/Glmhr1. **(B)** Western blotting analysis. M: Maker; 1: positive colony. **(C)** Determination of the content of lanosterol and ergosterol. 1: standard; 2: strain GS115; 3: strain GS115/Glmhr1.

A 1,732 bp DNA product of *Glmhr1* (GL25628) gene was obtained and ligated into the pPIC9k vector to construct the recombinant plasmid pPIC9k/Glmhr1, which was verified by *Xho*I and *Not*I digestion (Figure 5A). The linearized plasmid pPIC9k/Glmhr1 was transformed into *P. pastoris* GS115 to obtain recombinant strain GS115/Glmhr. Positive transformants were selected on MD plates and verified by PCR amplification. Western blotting revealed that the molecular weight of expressed Glmhr1 in recombinant strain GS115/Glmhr1 was about 74 kDa, which was consistent with the theoretical molecular weight (Figure 5B). In addition, the contents of lanosterol and ergosterol in GS115 were 261.5 mg/g DCW (dry cell weight) and 195.4 mg/g, respectively. While those in GS115/Glmhr1 were 163.7 and 194.7 mg/g, respectively (Figure 5C). The lanosterol content in GS115/Glmhr1 was significantly less than that of GS115, but there was no difference in ergosterol content between them suggesting that *Glmhr1* has a negative regulation effect on lanosterol production.

## Discussion

*G. lucidum* contains significant amounts of triterpenoids, which are bioactive compounds with antitumor and immune-modulatory properties. At present, more than 150 triterpenoids have been discovered in *G. lucidum*. Forty-eight and 40 triterpenoids were identified in the fruiting body of strain G203 and CICC 14022, respectively, but only a few were detected in both strains, such as Ganoderic acid A, D, F, G, H, and L, indicating that the GT composition varied depending on the growth stage and place of origin (Xie et al., 2020). In this study primordium and fruiting body have similar triterpene composition, but the contents of many components in primordium were higher than that in fruiting body. The amount and contents of triterpenes in aerial mycelia were lower than those in primordia and fruiting body, which is consistent with our previous study (Chen et al., 2012). Triterpenoids are important bioactive components and biosynthesized through the MVA pathway in *G. lucidum*. Some known genes associated with the MVA pathway had been reported to be upregulated in responsive to MeJA, including hydroxy-3-methylglutaryl-coenzyme A synthase (*HMGS*), hydroxy-3-methylglutaryl-coenzyme A reductase (*HMGR*), mevalonate-5-pyrophosphate decarboxylase (*MVD*), farnesyl pyrophosphate synthase (*FPS*), squalene synthase (*SQS*), and oxidosqualene cyclase (*OSC*) (Ren et al., 2010). Meanwhile, the yield of ganoderic acids was 45.3% higher than the untreated control sample after 254 μM of methyl jasmonate treatment (Ren et al., 2010). In this study, the content of total GTs reached the highest value at 200 μM, which was 2.17 times higher than that of the control group, also indicating that MeJA could increase the production of GT.

There have been some reports on the induction of the content of total triterpenoids using MeJA and other elicitors, only a few genes have been further described (Chuang et al., 2009; Zhang et al., 2010; Zhang and Zhong, 2013; Da et al., 2015; Sudheer et al., 2016; Feng et al., 2017; Gu et al., 2017). In *G. lucidum*, ozone gas and nitric oxide (NO) also had been reported to increase the content of GTs (Sudheer et al., 2016; Gu et al., 2017), and the transcriptome analysis of nitric oxide response had been reported. Besides, NO might decrease Acon activity by S-nitrosylation at Cys-594 to regulate GA biosynthesis under heat stress condition in *G. lucidum*. Previous research have described the mechanism by which elicitors the biosynthesis of triterpenoids, but few TFs were described in detail. Additionally, cDNA-AFLP was used to screen differentially expressed transcripts (TDFs) of the MeJA-treated mycelia of *G. lucidum* and to gain insights into the regulatory mechanisms of GA biosynthesis in response to MeJA. Only 90 TDFs were annotated with known functions through BLASTX searching of the GenBank database (Ren et al., 2013a). As previous reported, there were a total of 16,113 genes in the *G. lucidum* genome (Chen et al., 2012). The above studies have provided limited information about the function of TFs involved in the regulation of GTs biosynthesis.

Many TFs in plants have been extensively studied for their roles in secondary metabolites biosynthesis, such as, JA- or MeJA-responsive AP2/ERF, C2H2, MYC, MYB, HLH, and WRKY TFs and their functions have been described to improve both the production of metabolites and biomass, as well as upregulated the expression of genes (Tsubasa and Takashi, 2011; Zhang et al., 2011; Yu et al., 2012). However, TFs in fungi are more reported for their roles in the growth and development than in secondary metabolites biosynthesis, resulting in a limited understanding of the transcriptional regulation of filamentous fungi. In *G. lucidum*, *GlPacC* (GL370073) silencing inhibits the growth rate of mycelium, the development of the fruiting body and the synthesis of ganoderma acids (Wu et al., 2016). *Swi6*, which belongs to the APSES family, is a TF unique to fungi. Study have shown that the silencing mutant of *GlSwi6* (GL18755) in *G. lucidum* reduced the growth rate of mycelium, increased mycelium branches, inhibited the formation of fruiting body, and reduced the content of ganoderma acid (Zhang et al., 2018). Additionally, *GlSkn7*, is a highly conserved stress-responsive TF, whose silencing resulted in hypersensitivity to oxidative stress and the increase of the content of GAs (Wang et al., 2018). These transcription factors involved in mycelial growth are also involved in triterpenoids synthesis. In this study, 40 TFs were found to be correlated with the contents of triterpenoids (Supplementary Table 3), suggesting that they might be involved in the regulation of GTs biosynthesis.

Eight negatively regulated TFs were screened by MeJA treatment, and *Glmhr1* was proved to negatively regulate lanosterol synthesis. *Glmhr1* contained a fungal_ TF_ MHR domain functional domain, which is present in the fungal zinc cluster transcription factors that contain a GAL4-like C6 zinc binuclear cluster DNA-binding domain. In our previous study, *Glmhr1* was screened by yeast one-hybrid library system and considered to be a candidate TF for regulating the expression of *FPS*, which is the important gene involved in lanosterol biosynthesis (Xu et al., 2020). Some GAL4-like transcription factors, like GAL4 and STB5, has been extensively studied for their regulatory role in the metabolic process. *Gal4* is a transcriptional activator of genes associated with galactose metabolism. Yeast Gal4 recognizes and binds promoter UAS through its N-terminal DNA-binding domain, and initiates RNA polymerase II complex assembly and transcription by interacting with transcription factors through its C-terminal activation domain. *SUT2*, a Gal4-like gene in *Pichia pastoris*, whose overexpression could increase ergosterolcontent in cells (Yang et al., 2020). While, *STB5* is a negative regulator of azole resistance in *Candida glabrata*. Its overexpression resulted in the repressed azole resistance, and its deletion caused a modest increase in resistance (Noble et al., 2013). In this study, the overexpression of *Glmhr1* resulted in the decrease of lanosterol content, indicating that it may affect triterpene content of *G. lucidum* by decreasing the production of lanosterol. But how does *Glmhr1* regulate the lanosterol biosynthesis need further study.

HMG-box TFs were reported to play a great important role in fungal growth development. There are total of 20 HMG-box TFs in the genome, and 5 genes could be elicited by MeJA. Among them, GL31187 was highly expressed in primordia and fruiting body, especially in primordia. GL31187 was identified as *NHP6B* that was small and abundant chromatin proteins without sequence specificity for DNA binding (Stillman, 2010). In yeast, Nhp6A/B proteins, whose deletion mutant (nhp6DD mutant) is temperature sensitive for growth, were required for the activation of RNA polymerase II and the promotion of RNA polymerase III transcription (Costigan et al., 1994; Stillman, 2010). Several other fungal HMG-box TFs such as *pcc1*, *exp, hom1*, and *hom2* played an imprtant role in fruiting body formation and spore production (Ohm et al., 2011; Ait et al., 2013).

MADS-box TFs were also involved in the regulation of hyphal growth and sexual development. In the *G. lucidum* genome, both 2 MADS-box TFs were upregulated in response to MeJA treatment. Among them, GL23559 was highly expressed during the three growth periods, especially in the primordia, indicating its participation in all the growth periods. It was identified as a MADS-box TF homologous to TF *Mcm1* in *S. cerevisiae*, which regulated the expression of genes related to arginine metabolism, G2-specificd transcription and mating-type switching (Messenguy and Dubois, 1993; Wu et al., 1998). *Mcm1* homologous have also been characterized in the *Beauveria bassiana* and *Fusarium graminearum* (Yang et al., 2015; Zhao et al., 2019), which also involved in fungal development. Mcm1 homologous TF *SrfA* is required for pore differentiation through regulation of the expression of spore-specific genes involved in late events of spore maturation. And the *mcm1* gene deletion mutant resulted in increased hyphal branching, reduced biomass, and reduced hyphal compartment length during vegetative growth. The mutant strain was unable to produce a fruiting body or ascospores and microconidium during sexual development (Nolting and Pöggeler, 2006; Zhou et al., 2011; Qu et al., 2014).

In addition, there were some other MJ-responsive transcription factors that were also involved in fungal growth. Current researches revealed that bZIP TFs play an important role in response to biotic and abiotic stresses, regulation of growth and development, and biosynthesis of secondary metabolites (Balázs et al., 2010; Gai et al., 2022). There are 12 bZIP TFs in the genome, and 6 of them were upregulated in response to MeJA treatment. Among them, GL28195 was expressed in the mycelia, the primordia and the fruiting body at high level, indicating its participation in all the growth processes. In addition, GL26649 was identified as *Aft1*, which is a homolog to the gene isolated from *Saccharomyces cerevisiae* and *Schizosaccharomyces pombe*. *Aft1* plays a central role in iron homeostasis and interacts with an iron-responsive element (FeRE), affect the osmotic stress response (OSA), and also is necessary for sexual development in entry into the stationary phase (Berthelet et al., 2010; Miao et al., 2011). What’s more, GL30843 was predicted to contain a YAP domain similar to *S. cerevisiae YAP1*, which is required for tolerance of oxidative and osmotic stress (Gruhlke et al., 2017). The yapA deletion mutant exhibited delays in the rate of growth, germination, and conidiation. The same regulatory function in stress tolerance had been characterized in *Epichloe festucae* and *Aspergillus fumigatus* (Lessing et al., 2007; Cartwright and Scott, 2013). These bZIP genes properly played an important role in the growth period of *G. lucidum*.

The C2H2 TFs are one of the most ubiquitous transcription factor families in eukaryotes (Ren et al., 2013b; Yuan et al., 2018). In fungi, C2H2 TFs are involved in cell differentiation, mycelia growth, asexual reproduction and oxidative stress signaling pathways (Messenguy and Dubois, 1993; Jin et al., 2014; Malapi-Wight et al., 2014; Yao et al., 2016). In the *G. lucidum* genome, there are 79 C2H2 TFs. Among them, the C2H2 TF *PacC* (GL30073), the deletion of which would inhibit the growth of mycelia and the spore production (Wu et al., 2016), was not detected in this study. While its expression level in the mycelia, the primordia and the fruiting body was not high, indicating that some genes with low expression level are also indispensable in the growth period. In this study, 14 C_2_H_2_ DETs were upregulated and 1 downregulated in response to MeJA. GL26097, identified as *Sfp1* that has been reported as a stress- and nutrient-sensitive TF that regulates ribosomal protein gene expression in yeast (Marion et al., 2004). Deletion of the *Sfp1* gene resulted in slow cell growth and cell adhesion, indicating that *Sfp1* is required for normal fungal growth (Albert et al., 2018).

## Conclusion

In *G. lucidum*, the composition of triterpenes is different in different stages, and MeJA could induce an increase in triterpene concentration and biomass of mycelia. Transcriptome sequencing was used to analyze TFs in response to MeJA-induction, and a total of 103 DETs were identified. Furthermore, corresponding expression levels of these TFs in mycelia, primordia and fruiting body were analyzed. The results revealed that most of MeJA-responsive TFs extensive participation in the period of GTs biosynthesis and growth development. Furthermore, *Glmhr1* was verified to have a negative regulatory effect through decreasing the production of lanosterol.

## Data availability statement

The datasets presented in this study can be found in online repositories. The names of the repository/repositories and accession number(s) can be found in the article/Supplementary material.

## Author contributions

XX, SC, and JR conceived and designed the experiments. FZ, YL, and YZ performed the experiments. XX and FZ analyzed the data. FZ and HZ contributed to the reagents, materials, and analysis tools. XX wrote the manuscript. SC and JR revised the manuscript. All authors contributed to the article and approved the submitted version.
